# Shaping the future learning environments with smart elements: challenges and opportunities

**DOI:** 10.1186/s41239-021-00254-1

**Published:** 2021-03-15

**Authors:** Simon K. S. Cheung, Lam For Kwok, Kongkiti Phusavat, Harrison Hao Yang

**Affiliations:** 1 The Open University of Hong Kong, Hong Kong, China; 2 City University of Hong Kong, Hong Kong, China; 3 Kasetsart University, Bangkok, Thailand; 4 State University of New York At Oswego, Oswego, NY USA

## The evolving learning environments

Unarguably, technology has become an irreversible force driving the transformation of teaching and learning practices. Cloud computing, learning analytics, big data, and artificial intelligence are being adopted in today’s teaching and learning, though to different extents. For over a decade, educational researchers have been exploring how different innovative means could be integrated into traditional learning in order to enrich learning experience and enhance learning effectiveness. Enabled by various pedagogical and technological innovations, brand new learning environments can be created to optimize learners’ ability to learn. They are collectively referred as the commonly known “smart learning environments” which can best delineate the future learning environments. Embracing a variety of concepts, including but not limited to flexible learning, personalized learning, mobile learning, adaptive learning, and blended learning, for obvious reasons, there are no one single form of smart learning environments. The concepts, and even definitions, of smart learning environments have continuously emerging.

A smart learning environment can be conceptualized as a learning environment that emphasizes learning flexibility, effectiveness, efficiency, engagement, adaptivity, and reflectiveness (Spector, 2014), where both formal learning and informal learning are integrated (Gros, 2016). It is basically an adaptive system that improves learning experience based on learning traits, preferences and progress, features increased degrees of engagement, knowledge access, feedback and guidance, and uses rich-media with a seamless access to pertinent information, real-life and on-the-go mentoring with the use of technologies to continuously enhance the learning environment (Singh & Hassan, 2017). In recent years, educational researchers have been actively investigating a smart learning environment. As of March 2021, a simple search of the keyword, “smart learning environment”, from Google Scholar and Scopus yields 1990 and 1773 results respectively. Over 80% of these results are published within 5 years, and almost all refer to the tertiary education settings, including higher education, further education and open education.

This thematic issue entitled *Future Learning Environment: Pedagogical and Technological perspectives* aims to report the latest research findings and share good practices on creating brand new learning environments that emphasize learning effectiveness, efficiency, flexibility and engagement. It started to invite submissions in April 2020. By the submission deadline in December 2020, over 160 submissions had been received. After a rigorous and highly selective review process, 6 papers were finally accepted to this thematic issue. All of them are well written with significant original contributions that would provide excellent references for educational researchers and practitioners in the pursuit of the best learning environments.

Serving as the editorial introduction, this position paper first discusses the new challenges and opportunities in designing and implementing smart learning environments in the higher education context, and then highlights the key findings reported by each article appearing in the thematic issue.

## Current issues and new challenges

Today, the education system has been undergoing major changes brought about by emerging educational concepts and technological reforms. These emerging concepts and reforms pose a number of new challenges on smart learning environments, as enumerated below.

### Pedagogical approaches

Many advances in education will be brought about by the further integration of personalized learning and intelligent learning environments (Price, 2015). Chatti et al. (2010) pointed out that learning is personal, social, distributed, universal, flexible, dynamic, and complex. In a smart learning environment, a fundamental shift is needed towards a more personalized, social, open, dynamic, emergent, and knowledge-pull model for learning, as opposed to the one-size-fits-all, centralized, static, top-down, and knowledge-push models of traditional learning solutions (Chatti et al., 2010). To achieving this goal, new pedagogical approaches are required regarding the effective application of integrating technologies into the curriculum in a smart learning environment, to improve the effectiveness and efficacy of students’ learning.

### Personalized adaptive learning

In a smart learning environment, more attention has been paid to individual needs of students. According to Hwang and Fu (2020), a smart learning environment is regarded as a learning system for facilitating efficient personalized learning. Adaptive learning provides technical and methodological support for personalized learning. Personalized adaptive learning makes adaptive adjustments according to the individual characteristics of learners to promote the individualized development of students.

Smart devices and intelligent technologies in smart learning environments can be used to promote the development of personalized learning and adaptive learning for students. The smart learning environment has a large potential to effectively promote the development of personalized learning and adaptive learning (Peng, Ma & Spector, 2019). Thus, how to design learning ecosystems that integrate smart learning to personalize and self-regulated learning will be a key challenge (Gros, 2016). The following efforts could be made: monitoring learners’ differences and changes in individual characteristics, individual performance, personal development, and adapting teaching strategies (Peng, Ma & Spector, 2019).

### Affective interaction

New knowledge is constructed through social interaction. Just because the smart learning environment makes it technically possible, it does not mean that social interaction will necessarily occur (Feidakis, et al., 2013). Emotion is a kind of psychological response of human beings, which can influence and regulate cognitive activities such as attention, perception, representation, memory, thinking, and language. It occurs in the social interaction between students. In the traditional face-to-face learning environment, affective interaction occurred among teachers and students at a very high frequency, while smart learning environments focus more on imparting knowledge than affective interaction. Therefore, how to improve the affective interaction within the smart learning environment is an important challenge nowadays. One effective solution is to construct a comprehensive and dynamic learner model, which can incorporate learners’ learning emotions as a more important influencing factor (Hwang & Fu, 2020).

### Assessment method

Despite advances in psychological research and educational technology, assessment practices in educational institutions have remained unchanged for decades. Under a smart learning environment, there is an urgent need to go beyond traditional forms of assessment and use new methods to evaluate the effectiveness of the smart learning environment. The formative assessment might be an effective approach. It can enhance students’ ability to change from passive learners to active learners, where they can understand their strengths and weaknesses, recognize gaps in learning and develop solutions (Price, 2015).

### Integration of formal learning and informal learning

In the past, the channels for students to acquire knowledge were formal school and university studies, but now through the Internet, students can easily obtain and use informal learning methods, which leads to formal learning time allocation may only account for 50% of learners’ study time (Kinshuk et al., 2016). However, due to the blurring of the boundaries between formal and informal learning and the increasing attention to informal learning, the smart learning environment must integrate formal and informal learning to create an autonomous learning environment to support individual learners (Gros, 2016).

### Learning data

Education (in whatever form) has always used data (such as demographic and behavioral data) to plan, operate and teach, and smart technology offers new opportunities to extend the "data gaze" (Kwet & Prinslo, 2020). In a smart learning environment, a large amount of learner behavior data is generated. However, it is important to note that the data collected for these forms of delivery may vary depending on technology, background, institutional characteristics, and pedagogical strategies (Broughan & Prinsloo, 2020; Pink, et al., 2018). Therefore, how to integrate data in different scenarios, build data-centric smart education, and provide learners with a seamless learning experience and personalized customized services is also a big challenge (Zhu, Yu & Riezebos, 2016). It is also a challenge to collect and use these learning data, while observing relevant data protection principles and guidelines. Learning analysis could be used to process learning data, monitor learning progress, and provide feedback to the system, teachers, and students. However, the current studies on the design and implementation of learning analytics as reported in the literature are found to be largely driven by researchers in areas of computer science and decision sciences (Lee et al., 2020). The focus is on the applications of analytics to teaching and learning more from the technological perspectives that the pedagogical perspectives.

## Opportunities and development trends

In the last decade, innovations have emerged into teaching and learning practices at an ever accelerating rate. The latest advances in pedagogies and technologies have brought new opportunities on the development of smart learning environments in two aspects, namely, performance evaluation and instructional design. The following discusses these opportunities with suggestions.

### Evaluation of a smart learning environment

#### Evaluation on learning performance would be more accurate

Through artificial intelligence technologies in a smart learning environment, such as the internet of things, perception technology, video recording technology, image recognition technology and platform acquisition technology, multi-source, heterogeneous, multi-modal big data (for example, raising hands, facial expressions, bodily postures, and discussion) concerning with students’ learning process could be collected (Beer, 2019; Chatterjee et al., 2019; Kwet & Prinsloo, 2020). Such big data would generate new insights about students’ behavior and learning performance in the smart learning environment, which makes it possible to better understand and optimize the learning process and the teaching environments (Shorfuzzaman et al., 2019; Syafrudin et al., 2018). For instance, the “artificial intelligence smart classroom” solution by Intel partner Corerain utilizes video analytics to detect and identify students’ positive actions, such as participation, hand raising, and standing up, and negative actions, such as turning around and resting their head on the table. Then, these actions would be traced, recorded, and visualized in a dashboard to determine students’ engagement situations (Intel, 2019).

#### Feedback and intervention would be more timely

With the help of learning analytics, a smart learning environment could monitor students’ learning process, alert possible academic failures, conduct timely and effective interventions for learning problems, and provide students with personalized support services (Pardo et al., 2019; Tempelaar et al., 2021). Specifically, with the application of machine learning and predictive modeling techniques, learning analysis could help to identify students at risk of failure or dropping out, and provide special support, such as course recommendation, instructional design (Sclater, 2017; Xing, et al., 2019). For instance, in response to the declining freshman retention rate, Purdue University launched the course signal system, which could collect and analyze data, such as student course performance, learning behavior, previous academic history, learner characteristics, etc., to realize real-time prediction of a course. Since students’ performance would be indicated by different signals, teachers can appropriately intervene in students’ learning by sending emails, text messages, and face-to-face interviews according to the signals. Furthermore, teachers can also guide learners through recommending appropriate learning resources of the system to promote their success in learning (Arnold & Pistilli, 2012).

### Instructional design in a smart learning environment

#### Instructional resources would be more equitable

In a typical smart learning environment, digital cameras and recording or casting equipment, multiple student-controlled interactive whiteboards or touch screen televisions, mobile devices that are compatible to connect with student-controlled displays, wireless Internet, and educational management software are ubiquitously available (MacLeod et al., 2018). These equipped resources and technologies could ensure all students in a smart learning environment have the access to engage in different kinds of instructional resources regardless of race, gender, learning differences, socio-economic status, or background.

#### Instructional approaches would be more student-centered and flexible

With the help of smart technologies, existing researches have shown that active learning approaches, including inquiry learning, collaborative learning, group learning, and so on, are increasingly ubiquitous (Ellis & Bliuc, 2016). With the continued maturing of smart technologies, these student-centered instructional approaches could be more common. With the ability to store, collect, compute and analyze the massive data of learners to do the optimized pedagogical decisions (Li, Kong & Chen, 2015), a smart learning environment could push personalized learning plans for every student, at the same time, students could interact with the smart learning management system to adjust the learning plan. Besides interaction between students and the system, interactions between students and teachers, students and parents would be more convenient and timely, since the smart learning system could assist teachers in mastering students’ conditions and in adjusting teaching in real-time (Dai, 2019).

What’s more, the ubiquitous instructional resources in a smart learning environment make it possible for students to conduct any learning activities with their preferential learning approaches at anytime and anywhere they wanted (Hwang, 2014). Students could choose their classmates by themselves, some in a face-to-face environment whilst some others in the cloud. Compared with the fixed time and fixed classroom in the traditional instruction, the instructional approach in the smart learning environment would be more flexible.

#### Instructional objectives would be more ability-centered

Previous studies have also indicated that a smart learning environment can stimulate students’ learning motivation, promote active learning, improve academic performance and stimulate higher-order thinking skills (Jena, 2013; Liu et al., 2011; Lu et al., 2021). With the tendency that the instructional approaches to be more student-centered, with the interaction between students, teachers, parents, and learning system to be more convenient, students would have more free space to develop and conduct learning activities by communicating and collaborating with their classmates, or seeking help from their teachers. This active learning process can not only help students gain new knowledge, but also cultivate their cognitive, behavioral, and emotional skills.

As a final note, while learning environments continue to evolve, the learning process itself is inevitably undergoing different levels of transformation. It is also about time for the learning process to be reviewed or even re-defined.

## Papers in this thematic issue

The upcoming 6 papers collectively attempted to address the challenges and evaluate the effectiveness of learning environments, as well as to develop new instructional design approaches and technological measures.

The first upcoming paper entitled, “Past, Present, and Future of Smart Learning: A Topic-based Bibliometric Analysis”, provides a literature review of smart learning. The authors conducted a topic-based modelling analysis on the publications relevant to smart learning. The major research topics on smart learning were identified, for example, interactive learning, multimedia learning, STEM (science, technology, engineering, and mathematics) education, blended learning, affective and biometric computing. Some emerging topics, such as learning analytics, IoT (Internet of things), could computing, MOOCs (massive open online courses), and feedback and assessment, were also identified. The authors attempted to explain how these topics evolved over the years. The findings help educational researchers, practitioners and policy makers better understand the past, present, and future of the development of smart learning and smart learning environments.

The second paper entitled, “Technology Acceptance of Four Digital Learning Technologies (Classroom Response System, Classroom Chat, E-Lectures, and Mobile Virtual Reality) after Three Months’ Usage”, provides a reflection of how the four popular learning technologies are compared under a technology acceptance model. The study was carried out through a survey conducted to the students of a university in Switzerland. Three core factors, namely, perceived usefulness, perceived ease of use, and behavioural intention, were considered. The results showed that classroom response systems had the highest level of acceptance, followed by e-lectures, and then classroom chat, and then mobile virtual reality. The authors admitted that the low level of acceptance for mobile virtual reality was surprising and went contrary to their expected results. Feedbacks from students were studied, revealing a substantial drop in perceived usefulness and behavioural intention.

The paper to follow is entitled, “Transitioning to the New Normal of Learning in Unpredictable Times: Pedagogical Practices and Learning Performance in Fully Online Flipped Classrooms”. The authors shared their successful experience in transforming two conventional flipped classes into fully online flipped classes with the help of a cloud-based video-conferencing app, in order to cope with the immediate switching of classes to online delivery modes due to the COVID-19 outbreak. The transformation was explained, based on the 5E (Engage, Explore, Explain, Elaborate, and Evaluate) framework for flipped classes. The effect of fully online flipped classes on learning performance was evaluated. The results showed that the online flipped classroom approach can be as effective as the conventional flipped classroom approach. A number of good practices for using video-conferencing tools to support online flipped classrooms were proposed. Useful guidelines on the implementation of online flipped classes were provided for reference.

In the fourth paper entitled, “Examining the Key Influencing Factors on College Students’ Higher-Order Thinking Skills in the Smart Classroom Environment”, the authors conducted a structural equation modelling analysis to study the relationships between key factors that influence students’ learning and higher-order thinking skills in a smart classroom environment. It was revealed that peer interaction and learning motivation had a direct impact on higher-order thinking skills. Indirect effects were found between students’ learning strategy and higher-order thinking skills through the mediator peer interaction, and between smart classroom preferences and higher-order thinking skills through the learning motivation, the combination of learning strategy and peer interaction, and the combination of learning motivation, learning strategy and peer interaction. Accordingly, recommendations were made for teaching higher-order thinking skills in a smart classroom environment.

The next two papers shift the focus on improving the learning environments with technologies such as virtual reality and lecture capturing systems, where the benefits and advantages are illustrated.

The fifth paper entitled, “Benefits of Immersive Collaborative Learning in CAVE-based Virtual Reality”, demonstrated the use of immersive virtual reality in learning complex subjects for more engaging, motivating and effective learning experience. Taking neuroanatomy as an example of a visually and spatially complex subject, a virtual reality game was developed in a cave automatic virtual environment or CAVE for learning brain structures, their interconnections and broader spatial relationships. The game consisted of an interactive virtual learning environment which employed all four walls of a CAVE to provide an immersive and engaging experience to groups of learners. Constructivist elements, such as free exploration, knowledge construction and collaboration, were incorporated. It was found that learning in a CAVE yielded higher learning gains, as compared to the conventional textbooks, and that low spatial ability learners could benefit most from the strong spatial cues provided by immersive virtual reality in term of improvement in performance.

The last paper entitled, “Investigating the use of a lecture capture system within pharmacy education: Lessons from an internationally accredited undergraduate pharmacy program”, discussed the use of a lecture capture system to assist students in grasping difficult concepts. The authors conducted an analysis of 18 courses over three academic years. The results showed that year-1 students viewed lecture captures most frequently at the beginning of the academic year, followed by year-2 students, and then year-3 students, and that such pattern was further underscored by the class of 2020. Based on the findings, the authors proposed professional development for faculty to showcase the advantages of the lecture capture system and the benefits of a multitude of learning and teaching styles and methods, while also suggesting further quantitative and qualitative studies to help grasp the students’ motivations for use, and their attitudes and perceptions towards the system.

All these papers would contribute to help shape the future learning environments with various smart elements from both pedagogical and technological perspectives. We hope that you would enjoy reading the papers.
